# Treatment recommendations for non-HIV associated cryptococcal meningoencephalitis including management of post-infectious inflammatory response syndrome

**DOI:** 10.3389/fneur.2022.994396

**Published:** 2022-12-01

**Authors:** Kenneth Ssebambulidde, Seher H. Anjum, Jessica C. Hargarten, Prashant Chittiboina, Shmuel Shoham, Seyedmojtaba Seyedmousavi, Kieren A. Marr, Dima A. Hammoud, Bridgette Jeanne Billioux, Peter R. Williamson

**Affiliations:** ^1^Translational Mycology Section, Laboratory of Clinical Immunology and Microbiology, National Institute of Allergy and Infectious Diseases, National Institutes of Health, Bethesda, MD, United States; ^2^Surgical Neurology Branch, National Institute of Neurological Disorders and Stroke, Bethesda, MD, United States; ^3^Johns Hopkins School of Medicine, Baltimore, MD, United States; ^4^Microbiology Service, Department of Laboratory Medicine, Clinical Center, National Institutes of Health, Bethesda, MD, United States; ^5^Center for Infectious Disease Imaging (CIDI), Radiology and Imaging Sciences, Clinical Center, National Institutes of Health, Bethesda, MD, United States; ^6^Section of Infections of the Nervous System, National Institute of Neurological Disorders and Stroke, National Institutes of Health, Bethesda, MD, United States

**Keywords:** cryptococcal, *Cryptococcus*, infectious diseases, fungus, neuroinflammation

## Abstract

Cryptococcal meningoencephalitis (CM) continues to cause major morbidity and mortality in a range of patients such as those immunosuppressed from HIV and with biologic immunosuppressants, including treatments of autoimmunity, malignancies, and conditioning regimens for transplantation. It is currently the most common cause of non-viral meningitis in the United States. Infections in previously healthy patients also develop with autoantibodies to granulocyte-macrophage colony stimulating factor or with monogenetic defects. In all populations, mortality and significant long-term morbidity occur in 30–50% despite therapy, and immune reconstitution and post-infectious inflammatory response syndromes complicate management. To help with these difficult cases, we present here a practical tutorial of the care of a range of patients with CM in the absence of HIV/AIDS.

## Introduction

*Cryptococcus* is an opportunistic fungus which most frequently presents as a pulmonary infection or meningoencephalitis (CM) (1). CM continues to have significant morbidity and mortality in immunocompromised populations including those with HIV/AIDS as well as those immunosuppressed because of autoimmune diseases, hematopoietic malignancies, solid-organ transplants (2), as well as in previously healthy hosts without obvious immune suppression (1). HIV-related CM is the most common cause of global disease, with an estimated 152,000 cases and 112,000 deaths in 2020 (3, 4). However, in countries with greater access to medical care, CM has emerged as a common cause of non-viral meningitis, perhaps associated with increased use of biologic immunosuppressants (5, 6). Historically, up to a third of patients with CM do not have HIV or other recognizable causes of immunosuppression. Recent studies have described underlying risks associated with the presence of specific autoantibodies [ex., antibody against granulocyte-macrophage colony stimulating factor (GM-CSF)] and mutations, such as those in genes for GATA2 and NF-kappa-B essential modulator (NEMO) (7–10). The following summary is presented as a practical educational guide for the clinical approach to manage this diverse population of patients without co-existent HIV infections. These patients can be particularly complicated, due to a need for both microbiological and inflammatory control as well as the potential for multiple co-morbidities and, in transplant recipients, the possibility of having several simultaneous infections. An understanding of the pathophysiology in this select population contributes to a more rational therapeutic approach.

### The pathogen *Cryptococcus*

The fungus *Cryptococcus* is a basidiomycete yeast with over 30 species, but most infections are caused by two species, *Cryptococcus neoformans* and *Cryptococcus gattii*. *Cryptococcus neoformans* was historically divided into 4 serotypes, A–D, with *var. neoformans* and *var. gattii* distinguishing A and D vs. B and C, respectively; however, these have recently been recognized as two distinct species based on molecular sequencing. *C. neoformans* is divided into genotypes VNI and VNII and *C. gattii* is distinguished by 5 genotypes, VG1-V. There has recently been an attempt to give species names to all 7 genotypes (11), but this remains controversial (12). For clinical practice, the dominant clinical presentations and therapeutic management strategies can be appreciated between two species, *C. neoformans* and *C. gattii*, and these two designations will be utilized here.

*C. neoformans* is the main cause of infections in patients with immunosuppression, including HIV infection and related to pharmacologic therapies for underlying diseases. *C. gattii* is more commonly known to cause infection in people with no recognized immunosuppression (13). Both organisms are residents of soil, bird feces and decaying plant vegetation and can also act as plant pathogens against seedlings (14), but have the potential to infect a wide range of cold-blooded and warm-blooded species (15). Key to infecting such a wide range of hosts is their ability to adapt and evolve under the selection of hostile environmental conditions and defenses against both plants and phagocytic predators such as amoeba and insects (14, 16). Historically, *C. neoformans* was felt to be a predominant infection of the immunocompromised and *C. gattii* that of immunocompetent patients; however, more recently, *C. gattii* infections have been strongly associated with the presence of immunosuppression mediated by an autoantibody to granulocyte-macrophage colony stimulating factor (GM-CSF) (13).

### Clinical presentation: Signs and symptoms

One of the major risk factors of a poor outcome in CM is a delay in diagnosis (17, 18). An extreme example seen recently at our institution was a patient admitted to a psychiatric hospital for depression who underwent a diagnostic lumbar puncture (LP) only 24 months after his initial presentation. Highly immunocompromised patients can often present with multiple simultaneous infections, which may delay the diagnosis of cryptococcosis. In one such solid organ transplant patient, it was difficult to discern whether persistent lower respiratory tract and cutaneous infections that were considered bacterial in origin were actually due to *Cryptococcus*. A third previously healthy patient seen recently at our institution was treated for migraines over several months and developed worsening headaches after a COVID-19 mRNA vaccine, prompting a diagnostic LP which grew *Cryptococcus neoformans*. The worsening symptoms were likely due to the pre-existing fungal infection, exacerbated by an inflammatory responses from SARS-CoV2 vaccination reported in other organ-specific inflammatory disorders such as myocarditis (19). While headache is a prominent presenting feature in people with CM, regardless of the source of immunosuppression, CM in previously healthy individuals without obvious immunosuppression frequently presents without fever (2). In the absence of fever, complaints of headaches and fatigue are presumed to be from other more common conditions, including sinusitis, migraines and even depression. However, in those with underlying pharmacologic immunosuppression (ex., solid organ transplant recipients), fever and headache are more common and, combined with the risk factors of immunosuppression, the diagnosis of cryptococcosis is typically considered and established earlier, resulting in improved outcomes in this population (20). In those without fever, secondary complications of elevated intracranial pressure may suggest more serious entities including cryptococcal disease. For example, visual changes can be a result of increased intracranial pressure acting on the optic nerve by venous congestion (21) or by direct invasion of the optic nerve sheath (22). Diplopia, especially that caused by CN-VI palsy is also a common presentation (23). Auditory symptoms that can either be unilateral or bilateral include loss of hearing or a “whooshing” sound and can be associated with inflammation of CN-VII or the internal auditory canal (24). Gait abnormalities are also common and may be the result of an accompanying spinal arachnoiditis or hydrocephalus (25). Evidence of a subcortical dementia with reduced executive and psychomotor function on detailed neuropsychiatric exams may also suggest cryptococcal disease (26), as distinguished from the frontal dementias typical of Alzheimer's.

### Imaging

Brain CT imaging at CM presentation is often unremarkable as small cryptococcomas are often not visible and inflammatory lesions are not well visualized (27). MRI can often be revealing, especially with sequences such as post contrast 3D-T1 weighted images which can show abnormal ependymal enhancement (ependymitis) as well as choroid enlargement and increased enhancement (choroid plexitis), when present. MRI post-contrast FLAIR sequences are particularly important for detection and follow up of meningeal enhancement (reflecting meningeal inflammation) which is not well visualized on post contrast T1 weighted images (28). Diffusion-weighted imaging can help identify ischemic events in the basal ganglia, along the distribution of small penetrating lenticulostriate arteries. In certain situation, space occupying cryptococcomas might show restricted diffusion as well. Those however can generally be differentiated from ischemic foci based on progression of diffusion restriction abnormalities (29). Cryptococcomas can sometimes show rim enhancement due to associated inflammatory reaction, however such lesions are unlikely to be confused with bacterial abscesses, based on history and imaging characteristics. MRI imaging can also be normal in up to 8% of HIV-related disease and up to 13% in non-HIV related disease (28–31). Elevated intracranial pressures with non-communicating hydrocephalus due to choroid plexitis, adhesions and secondary obstruction at the levels of the foramina of Monroe or Lushka/Magendie could result in entrapment of the ventricles or ventricular portions, with secondary transependymal CSF seepage and mass effect. We have not seen such entrapment in patients with HIV-associated cryptococcosis. Instead, the latter tend to have communicating hydrocephalus likely due to outflow obstructions from either inflammation or fungal organisms within the superior arachnoid granulations (32). Communicating hydrocephalus can also be seen in people who have CM without HIV. The importance of radiographic imaging is heightened in people who have CM without HIV, as central obstruction due to the increased incidence of choroid plexitis and ependymitis in these patients (28) can increase risk for uncal herniation when LPs are performed.

### Laboratory studies

Lumbar puncture with sampling of cerebrospinal fluid (CSF) is an important diagnostic tool. Diagnostic tests to detect cryptococcal capsular galactoxylomannan antigen in serum and CSF have been available commercially for over 2 decades, including latex agglutination-based antigen system (LA), the enzyme immunoassay-based assay (EIA) and the newest format as a lateral flow assay (LFA). Most of the initial studies were conducted in people living with HIV/AIDS (PLWHA) where all tests performed well (Table 1) (34–36). The LFA to detect cryptococcal antigen (CRAG) has provided better sensitivity than LA and this increased sensitivity is crucial to detect lower antigen loads that can be present in people who have CM with pharmacologic (non-HIV) or no obvious immunosuppression (36, 37). In one recent study, a LFA cryptococcal assay of blood was sufficient to diagnose cryptococcal disease in 28 previously healthy people who were found to have CNS disease (*n* = 21) or isolated pulmonary infection (*n* = 7) (37). In contrast, the EIA and the recently developed nucleic acid detection meningitis/encephalitis assays appear to be less sensitive but retain good specificity (38, 39). However, skin biopsy and/or culture can be positive in the absence of detectable blood antigens (40). Repeat antigen testing is also recommended when disease is highly suspected although repeatedly negative tests have a high negative predictive value. Notably, the utility of antibodies to detect *Cryptococcus* in patients with cryptococcosis is limited as the polysaccharide capsular antigen may inhibit the synthesis of antibodies (41). In addition, cross reactivity of cryptococcal antibodies in the CSF has also been noted for anti-*Histoplasma*, anti-*Coccidioides* and anti-*Blastomyces* antibodies in non-HIV infected patients with CM (42).

**Table 1:** Currently available antigen-based diagnostic assays to detect cryptococcal infections (33).

	**Test**	**Available methods**	**Preferred specimen**	**Performance**		
					**Sensitivity %**	**Specificity %**
*Cryptococcus neoformans /gattii* species complex	Antigen	LA, LFA, EIA	Serum	EIA and LA	83–97	93–100
				LFA	98–100	98–100
			CSF	EIA and LA	93–100	93–98%
				LFA	99–100	99–100

BAL, bronchoalveolar lavage; CSF, cerebrospinal fluid; EIA, enzyme immunoassay; LFA, lateral flow assay; LA, latex agglutination.

In addition to fungal culture of clinical specimens, Grocott's methanamine silver stain (GMS) can improve sensitivity of diagnosis and readily available stains such as mucicarmine and Fontana-Masson (FM) have specificity for *Cryptococcus* vs. other fungi such as *Candida* or *Blastomyces* (43). Staining of tissue specimens is particularly important in non-disseminated skin or bone disease (40, 44) Mucicarmine is a commonly available histochemical stain that, in addition to staining acid mucins of tumors, also stains the polysaccharide capsule of *Cryptococcus* spp. The FM stain is thought to demonstrate the presence of melanin in *C. neoformans* and *C. gattii*. This is particularly useful for strains of *Cryptococcus* that have a diminished capsule, which may not be readily apparent with the mucicarmine stain. In the setting of apparently local disease; however, lumbar punctures remain important to assess for neurologic dissemination from lytic bone lesions (45) or skin lesions (46), as the organism has a strong neurological predisposition for infection.

### Antifungal therapy

Much of the therapeutic recommendations for treatment of CM in people with pharmacologic immunosuppression have been derived from clinical studies of people with CM developing in context of HIV-related immunosuppression. Fungicidal therapy including amphotericin B formulations are a main-stay of therapy in cryptococcal meningitis whereas fungistatic therapies such as fluconazole at standard doses such as 400 mg daily are associated with poor outcomes and an inability to clear the fungus (47). There are also concerns about the emergence of fluconazole resistance (48), including heteroresistant subpopulations within a given infection (49) although controversy persists about the clinical significance of *in vitro* testing (50). In much of the world, liposomal preparations (L-AmB) are preferred because of less toxicity and more efficacy than deoxycholate formulations (51). The Infectious Disease Society of America (IDSA) recommends that people without HIV coinfection receive longer antifungal therapy; 4–6 weeks or 2 weeks after CSF culture negativity (52). Adjunctive 5-flucytosine is beneficial when given for at least 2 weeks in combination with amphotericin B, as shown in PLWHA (53). Recently, single high dose (10 mg/kg) L-AmB followed by high dose fluconazole (1,200 mg per day) + flucytosine (100 mg/kg daily) has proven to be effective in PLWHA CM patients but has not been studied yet in patients without HIV (54). After completion of induction therapy, patients are treated typically with 400–800 mg daily of fluconazole for extended periods though little data is available for treatment duration in people receiving pharmacological immunosuppression; durations can vary depending on the given dynamics of immunosuppression.

## Common scenarios affecting clinical response

### Microbiological control

Microbiological control, defined as achievement of negative CSF fungal cultures in CM, is a major prognostic factor in establishing a clinical response in HIV-related immune suppression as well as in non-HIV patients (47, 55, 56). The presence of renal and hematological toxicity may be significant in these patients (57), which may require interruption of therapy (58). An inability to continue with azole consolidation therapy is also a significant risk factor for recurrence based on HIV-infected populations (59–63). Indeed, in the pre-azole era prior to HIV, up to 15% of patients had recurrence of their infection despite achieving negative CSF fungal cultures by the completion of therapy (58). It is typically recommended to perform LPs at the 2-week mark of antifungal therapy, with termination of polyene antifungals 2 weeks after negative CSF fungal cultures. We follow HIV-related guidelines of continuation of fluconazole at 400–800 mg daily after completion of amphotericin induction therapy. These guidelines suggest 800 mg daily for consolidation if patients have not achieved sterilization of CSF cultures or have not improved clinically (64). In ambiguous cases, such as an inability to obtain CSF due to spinal obstruction or during the initial evaluation of a clinical failure, reference to minimum inhibitory concentrations (MICs) may be useful; relevant to this issue, *C. gattii* isolates may have higher MICs to fluconazole, especially those from the Pacific Northwest that are commonly VGII (65), prompting the use of the higher dose of fluconazole (800 mg daily) recommended by the IDSA guidelines (52) or next generation triazoles, voriconazole or posaconazole, or isavuconazole which have lower MICs to these strains (66, 67), although posaconazole is known to have poor central nervous system (CNS) penetration during intracranial fungal infections (68). In non-CNS infections, enlarging lung lesions could signal microbiological failure, in which case significant, progressive increments in serum cryptococcal antigens would be observed. If repeat biopsy is conducted it is important to obtain fungal cultures as the presence of intact organisms, much like the presence of persistent serum or CSF cryptococcal antigens, do not distinguish between live and dead organisms.

### Increased intracranial pressure

Increased intracranial pressure is an important prognostic factor for mortality with management implications and opening pressure should be obtained with every LP (69). It provides a risk assessment, especially as it pertains to the risk of visual loss and the need for repeated spinal taps for pressure management and even neurosurgical intervention. Recommendations derived from the HIV-literature are to perform repeated lumbar punctures for pressures above 250 mm H_2_O or symptoms of elevated intracranial pressure (70), which has been applied to the non-HIV population as well. Recently, one study suggested at least one additional opening pressure measurement to detect those who develop intracranial pressure elevations during induction therapy (71). Temporary extra-ventricular drainage devices (EVDs) can also be useful as monitoring and draining devices that can be used prior to definitive therapeutic drains including ventricular peritoneal shunts.

### Post infectious inflammatory response syndromes

Immune reconstitution inflammatory syndrome (cIRIS) is a common complication of CM in PLWHA, typically resulting from antiretroviral therapy (ART) supporting reconstitution of immunity, with a compartmentalized CNS inflammatory syndrome despite negative CSF fungal cultures (72). cIRIS has also been reported in transplant recipients which may also be a result of immune reconstitution if transplant-related immunosuppression is decreased in order to facilitate fungal clearance [see review, (73)]. In cryptococcal infections in those previously healthy, development of a similar paradoxical inflammatory syndrome with negative CSF fungal cultures has also been identified (74). In this case, lack of significant immune reconstitution led to the name post-infectious inflammatory response syndrome (PIIRS) (75). In PIIRS, immune presentation of intracellular proteins and cell wall constituents, released after cellular lysis from fungicidal therapy and/or after prolonged infection, results in compartmentalized CNS inflammation. This consists of increased accumulation of activated CD4 and CD8 cells, measured by the presence of HLADR^+^ CD4^+^ and CD8^+^ T cells in the CSF (74). Recruitment to the CNS appears dependent on chemokines such as CXCR3 in both human and mouse studies (76). In addition, monocytes are recruited to the intrathecal compartment, which appear on biopsy and autopsy studies to be in some cases non-phagocytic and non-fungicidal, representing alternatively activated macrophages as indicated by the expression of CD200R1 without inducible nitric oxide synthase (iNOS) (74). Soluble markers such as proinflammatory cytokines in the CSF are also elevated, particularly IL-6 as well as released products of T-cell activation such as sCD25 (74). In transplant recipients, physicians sometimes reduce immunosuppressive therapy in the setting of a serious fungal infection, in which case, a true immune reconstitution occurs, complicating therapeutic approaches (77). In such cases, it is important to balance the need for immune competency for microbiologic control vs. inflammatory modulation and organ maintenance, and several biomarkers and MRI studies described below may be helpful. In both types of immune activation, reductions in cryptococcal polysaccharide during antifungal therapy that have anti-dendritic and lymphocyte activity may also contribute to immune reconstitution (78, 79). Other promising approaches have sought to directly remove circulating immune-inducing antigens by CSF catheters but this remains a highly experimental approach at this time (80).

Clinically, PIIRS presents with either a deterioration in clinical status or a failure to improve in the setting of effective microbiological control, the latter demonstrated by negative CSF fungal cultures. The syndrome was defined in previously healthy patients (75) as shown in Table 2; and includes the Montreal cognitive assessment test (MOCA) where a score <22 was previously associated with poor outcome in a cohort of predominately transplant recipients and those previously healthy (2) as well as potentially irreversible complications including visual and hearing impairment and gait abnormalities, all in the setting of negative CSF fungal cultures. Historically, treatment recommendations of non-HIV patients in the setting of clinical deterioration following effective antifungal therapy included consideration of immunostimulants such as recombinant IFN-γ, which were based on HIV-related paradigms where poor microbiological clearance (56) and immune suppression associated with low CD4^+^ T cell counts are associated with susceptibility to CM (56). In the HIV setting, lower levels of IFN-γ could be an etiology for poor microbiological control and is a predictor of cIRIS after ART (81). However, robust accumulation of IFN-γ producing CD4^+^ cells within the intrathecal compartment in PIIRS, even in patients with systemic idiopathic CD4 lymphopenia, (74) do not support this paradigm in the majority of cases; PIIRS thus clearly adds complexity to the therapeutic equation and requires additional monitoring beyond microbiological parameters.

**Table 2:** Criteria of PIIRS (75).

**Main criteria**
1.Unchanged or declining mental status/cognition
2.Visual deficits not refractive in nature
3.Hearing changes
In a previously healthy patient with CSF fungal culture conversion to negative after initial amphotericin-based treatment regimens
**Supportive criteria**
1.Elevated CSF WBC and protein, and reduced CSF glucose
2.Increased CSF inflammatory markers i.e., IL-6 and soluble CD25 levels
3.Elevation in CSF activated immune cells (HLADR^+^ CD4^+^ T cells, HLADR^+^ CD8^+^ T cells, NK cells, monocytes
4.Abnormal brain and spinal cord MRI findings on post-contrast FLAIR showing but not limited to leptomeningeal enhancement, choroid plexitis, ependymitis, parenchymal lesions, hydrocephalus, arachnoiditis)

CSF, cerebrospinal fluid; WBC, white blood cell count; IL-6, interleukin 6; CD25, cluster of differentiation 25; MRI, magnetic resonance imaging; FLAIR, fluid attenuated inversion recovery.

### Diagnosis of PIIRS and preliminary testing for possible adjunctive immunotherapy

CSF fungal cultures as well as immunological tests including CSF flow cytometry and cytokines, particularly IL-6 and sCD25, are useful for the diagnosis and management of PIIRS (82). Negative CSF fungal cultures are necessary conditions for a diagnosis if PIIRS and are a reliable indicator of microbiological control, but do not prove fungal cure, necessitating consolidation oral antifungal therapy, especially if immunomodulatory therapy is contemplated. CSF total cell counts, if performed accurately, and total protein levels can also reflect relative inflammation, although it is important to remember that much of the inflammation occurs within the brain parenchyma, with CSF representing only a small proportion of the total intrathecal inflammatory burden (83). In the research setting, flow cytometry of freshly obtained CSF is useful, with absolute numbers of HLA-DR^+^ CD4^+^ T cells useful both to support the diagnosis and facilitate management (82). Soluble CSF cytokines can also be supportive of PIIRS and are available through commercial reference laboratories. In addition, lower CSF glucose concentrations are also indicative of more severe disease (74, 84). Since much of the accompanying intrathecal inflammation is compartmentalized within the CNS (74), ancillary testing of serum C-reactive proteins and D-dimers are not particularly reflective of CNS inflammation but may be useful to detect co-morbid extra-neural complications including pulmonary or urinary infections as well as deep venous thromboses, respectively. Since no data are available regarding corticosteroids at the initiation of antifungal therapy and may add increased risk, PIIRS should not be considered at the initial presentation while fungal cultures remain positive without further studies. In addition, it is important to assess for additional CSF infections using CSF screening tests for encephalitis or specific viral testing such as for HSV, VZV, EBV, JC, and CMV, as well as conditions that may increase risk of corticosteroids and require prophylactic therapy including hepatitis A, B, and C, and tuberculosis (TB) by QuantiFERON testing (Table 3). Baseline studies should also include eye examinations including visual fields, auditory testing, and MRI of brain +/– contrast agents with post-contrast FLAIR sequences, as well as MRI of the spine with contrast if symptoms suggestive of spinal arachnoiditis are present, such as fecal/urinary incontinence, elevated post-void residual volumes (25). In our experience, post-contrast FLAIR MRI images are much more sensitive than standard post-contrast T1-weighted images and are highly recommended for both diagnosis and follow-up of neuroinflammatory conditions.

**Table 3:** Studies prior to or in the early stages of initiation of pulse-taper therapy.

**Blood**
HIV, Hepatitis A, B, and C serologies
CD4 counts
CRP
TB QuantiFERON
**Cerebrospinal fluid**
Opening pressure, WBC, protein, glucose
IL-6 and soluble CD25
HSV, VZV, EBV, JC, and CMV PCR
Fungal culture
**Imaging**
Brain and spinal MRI
Bone density scan
**Physical**
Eye exam including visual fields
Auditory exam
Montreal cognitive assessment (MOCA)

### Initiation of adjunctive corticosteroid therapy

Recently, a prospective observational trial of adjunctive salvage therapy in PIIRS demonstrated effective clinical responses with pulse-taper corticosteroid therapy (PCT). This study of consecutive patients with PIIRS demonstrated favorable responses in virtually all patients after 1 week of high dose methylprednisolone (1 gram/day) followed by oral prednisone 1 mg/kg daily, with improvements in mental status (measured by MOCA and Karnofsky scores), visual exam, and hearing (82). We find that treatment of PIIRS typically is most successful if conducted within 8 weeks from CM diagnosis and antifungal initiation, and minor improvement is seen if initiated after several months, likely because of permanent scarring and irreversible injury at prior sites of inflammation. Patients are maintained on fluconazole at 400–800 mg daily consolidation therapy for at least the duration of immunosuppression as well as prophylactic trimethoprim/sulfamethoxazole 3 times weekly for prophylaxis of *Pneumocystis jiroveci* infections until the dose of oral corticosteroid is tapered to below the equivalent of 20 mg of prednisone per day (85). In addition, we have begun to add prophylactic valacyclovir to patients after several instances of herpetic infections in patients receiving prolonged corticosteroid courses for other diseases prior to transfer to NIH. This is especially important in the previously healthy population who may have as yet undiagnosed genetic defects or autoantibodies. Assessment for bone mineral density is also recommended (usually at discharge) because of the risk of osteopenia with corticosteroid therapy and guidelines in their use is available (86). This regimen of adjunctive PCT with fluconazole has not been associated with re-emergence of positive CSF fungal cultures in our experience despite ~60 patients being treated with various doses of corticosteroids at the NIH Clinical Center, although one should carefully monitor patients using serum cryptococcal antigens and CSF studies (including fungal culture) as indicated since an individual patient may have unique susceptibilities to recurrence.

**Figure 1:**
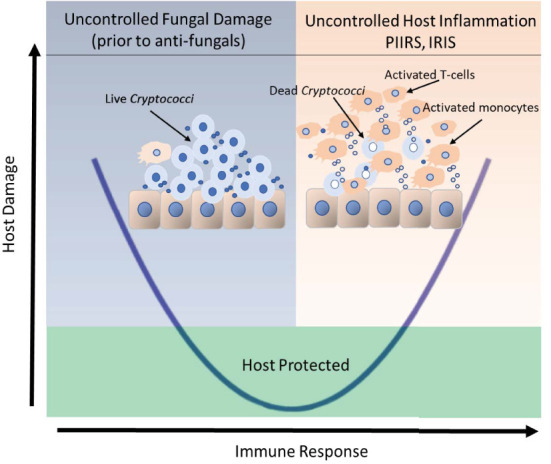
Clinical outcomes of host-cryptococcal interaction depicted by the basic parabola of the damage-response framework. The left side of the parabola, shaded in blue, depicts the historical concept that the live fungus was the primary contributor to host damage in the setting of a weak or normal immune response prior to therapy. The presence of an extracellular immune-inert capsule shields the fungus from detection by the host immune system. The right side of the parabola, shaded in orange represents an uncontrolled host response that may occur in response to increased fungal antigens released after antifungal therapy fungal lysis in the presence of (1) immune reconstitution after ART, (2) reductions in immunosuppression or (3) a relative intactness of the host immune system to unencapsulated released antigens in the previously healthy individuals. Uncontrolled host inflammation results in intrathecal recruitment of activated T-cells and monocytes and secondary cerebral edema, the latter of which contribute significant morbidity and mortality to the brain, which is confined by the bony skull. Uncontrolled cerebral swelling may increase overall intracranial pressure causing papilledema and loss of vision or life-threatening uncal herniation. The portion of the parabola that rises above the green-shaded box represents the threshold for clinical disease. The portion of the parabola that lies within the green box represents an effective yet balanced host control of the organism. Adapted from Pirofski and Casadevall (90).

Corticosteroids have known efficacy in a number of neurological conditions in that they both suppress adaptive immunity (87) as evidenced by reductions in CSF HLADR^+^ CD4^+^ T cells (and total CSF WBCs) by about 10-fold (82) as well as effects on vasogenic edema suggested by improvements in MRI imaging which could facilitate reductions in CSF opening pressure observed with PCT (88). This steroid efficacy is similar to the experience in cIRIS patients who also have inflammatory sequelae despite negative CSF fungal cultures after immune reconstitution with ART; however, the same efficacy is not seen in HIV-related immune suppression with CM at the initiation of therapy prior to ART, and may be deleterious (89). This suggests that damage during the acute treatment period is primarily due to fungal-related damage and may be augmented by immunosuppression by corticosteroids, whereas damage in cIRIS and PIIRS is related to immune-mediated damage and will likely benefit from immunosuppression. These two aspects of host damage have been represented by a parabola where too little immune response results in increased damage by the pathogen, and too much inflammation in cIRIS and PIIRS results in host-mediated immune damage (Figure 1). Each presentation requires separate measurements and requires specifically directed approaches and monitoring as below. For example, treatment of PLWHA with CM at HIV/CM diagnosis, where patients are presumably at the left hand side of the parabola, do not benefit from corticosteroid therapy, because host damage is the result of the fungus (89). A parallel model might be that of SARS-CoV2 infections, where anti-viral therapy is most effective early in disease, with immunosuppressives including corticosteroids reserved for the latter, inflammatory portion of the disease (91). Overall treatment summary is provided in Table 4.

**Table 4:** Treatment summary.

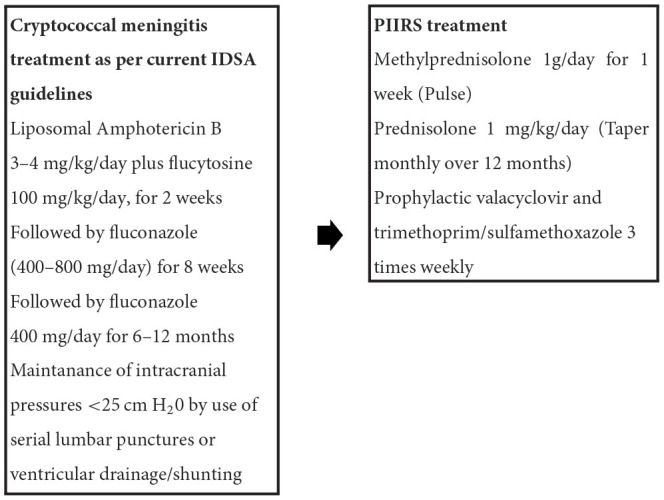

### Monitoring during acute therapy for PIIRS

Close monitoring during the initial period of PIIRS-directed therapy is essential. We typically perform an LP prior to initiation of PCT to provide a pre-treatment baseline and on day 7 at the conclusion of the pulse to monitor response, checking CSF opening pressures, fungal cultures, as well as commercially available parameters including total protein, total cell count, glucose, and cytokines including sCD25 and IL-6. One should see an approximate 10-fold reduction in CSF cytokines, a prompt reduction in cell count and a rise in glucose. CSF protein tends to lag improvements in other parameters. PCT has also been associated with reductions in opening pressures by a modest 120 mm H_2_O, which has obviated the need for shunting in some individuals (82). Importantly, cryptococcal infections do not represent a contraindication for either temporary or permanent ventricular shunting as the organism does not form significant biofilms under these conditions, and we have not seen indications of prolonged fungal culture positivity after shunting. Indeed, after shunt insertion, corticosteroid therapy typically reduces inflammation and debris, reflected in the CSF total protein, which may prevent proximal obstruction of the ventricular catheter (92).

Another important complication of PIIRS in the acute setting is the development of an inflammatory arachnoiditis in a significant proportion of patients (25). This can best be diagnosed by an MRI spine with contrast which can show meningeal enhancement of the conus and cauda equina neve roots in the acute stage as well as nerve root adhesions and clumping in the chronic stage. Clinically, this may be suggested by gait abnormalities such as a wide-based gait, lower extremity weakness or numbness, shooting lower extremity pains, and urinary obstruction with associated urinary tract infections, the latter tested by a post-void bladder scan or catheterization. Treatment is directed at the inciting inflammation similar to the central PIIRS process with corticosteroids. Seizures are another complication of CM and are more common after VP shunting or may be a harbinger of other complications such as venous sinus thromboses or secondary viral infections and warrant appropriate EEG, MRI and CSF studies. Furthermore, dehydration and immobility from mental status changes are risk factors for venous thromboses and a low threshold should be maintained for testing by leg and arm venous dopplers for peripheral thromboses or brain MR venography (MRV) for sagittal and transverse/sigmoid sinus thromboses.

Complications of corticosteroid therapy are also to be expected and include steroid-induced psychosis, elevated blood sugars and gastrointestinal (GI) complications. For patients with psychosis, we typically have a standing order for an anti-psychotic at bedtime plus additional prn orders as needed, being careful to select agents with minimal additive effects on QTc prolongation with that of azole antifungals. Psychiatric consult and EKG monitoring may be useful in such circumstances. Glucose monitoring and GI prophylaxis with proton pump inhibitors should also be implemented.

### Monitoring during follow-up therapy of PIIRS

Typically, one begins to see clinical responses in about 3 days, which continue to improve slowly but persistently over approximately a year. This distinguishes the condition from ischemic events, which typically plateau around 6 months, although may be influenced by the types of measurements used (93). Those older than 70 years or outside the 8-week window from initial CM diagnosis typically respond much more slowly. We typically discharge the patient on prednisone 1 mg/kg/day and follow up patients at 1-to-2-month intervals, using the MRI post contrast FLAIR as well as blood cryptococcal antigens along with CSF examinations with cytokines if ambiguities arise. The post-pulse CSF cytokine levels are helpful to establish a baseline level of inflammation to target if patients do not progress or exhibit clinical flairs. We will typically reduce prednisone doses over a period of a year at a rate of about 5–10 mg daily per month, much slower than that for cIRIS or TB meningitis (94). Despite this slow taper, immunological flares are common, typically manifesting as deteriorations with a similar (but not as severe) clinical profile and anatomical distribution as the primary manifestation. Though uncommon, we have seen clinical and immunological flares as far as 2 years out from initiation of therapy, requiring continued vigilance. MRI post-contrast FLAIR studies are particularly helpful to confirm the immunological flares and in ambiguous clinical situations. Lumbar punctures with CSF studies can also be helpful and negative CSF fungal cultures are useful to confirm that any clinical deteriorations are due to inflammation and not a recurrence of cryptococcal organisms. We typically treat flares with a “mini-pulse” of oral prednisone 30 mg/day above the previous dose for 3 days, followed by continuation at a dose 10–20 mg/day above that which the patient had been on prior to the flare followed by a re-establishment of the corticosteroid taper. Clinical responses to mini pulses are typically seen within 3 days. Fungal recurrences can also be monitored by blood and CSF fungal antigens if the laboratory is able to quantify the antigen load in a reproducible fashion. We have yet to see a fungal recurrence after initiation of corticosteroids; however, our experience is small (~60 patients) so vigilance for recurrence should still be high. Since the latex agglutination assay is less sensitive than the LFA (37), it is important to use one assay as switching between these two at different laboratories may cause confusion. Typically, a 4-fold increase in titer has been associated with a microbiological relapse but persistence of antigens or even visible organisms has not been shown to be associated with recurrences; rather, CSF fungal cultures remain the gold standard of microbiologic control. It is also important to realize that some late clinical deteriorations may not be from inflammation or the fungus and may simply be due to progressive scarring causing central or spinal obstruction. In this case, fungal cultures will be negative, inflammatory parameters (CSF cell counts and cytokines) and MRI post contrast FLAIR will not be consistent with inflammation. In this case consideration of ventriculoperitoneal shunting for elevated pressures that cause symptoms or risk visual loss may be indicated if present in consultation with neurology and neurosurgical advice. Additionally, repeat monitoring for late complications of steroid use is useful, including DEXA scans for bone mineral density reduction and eye exams for evaluation of posterior subcapsular cataracts (86). Rheumatological consultation may be helpful to manage chronic corticosteroid use.

### Special populations

Management of cryptococcosis presents special problems in solid organ transplant recipients. A major underlying risk for infection in such patients is their immune compromised state due to anti-rejection medications, particularly glucocorticoids. Use of non-calcineurin-based inhibitors is also a risk factor for both acquisition and poor outcome in cryptococcal disease (95). Effective fungal killing is assisted by reducing the net state of immunosuppression. However, for many patients there is a limited range of permissible modulation of immunosuppression before organ rejection and reductions may lead to over exuberant inflammatory response syndromes at the site of infection (96). Discontinuation of the calcineurin inhibitor (e.g., cyclosporin or tacrolimus) is especially associated with development of inflammatory response syndromes in such patients (97). Thus, in managing the balance between organ rejection and inflammatory syndromes of *Cryptococcus*, we have found that monitoring inflammatory parameters in the CSF such as IL-6 or sCD25 levels and imaging such as MRI post-contrast FLAIR images are particularly useful to modulate immune modulation therapeutics in addition to CSF fungal cultures. Treatment with amphotericin B, with its attendant nephrotoxicity, presents another challenge for solid organ transplant recipients. While amphotericin B is indispensable in the initial phase of treatment, if at all feasible, deoxycholate amphotericin B should be avoided, especially in kidney transplant recipients (98). Lipid formulations of amphotericin B are preferred; although less nephrotoxic, their use may still damage renal function and potentially lead to renal allograft loss. While only studied in HIV-related CM thus far, the use of high dose fluconazole in concert with flucytosine after loading doses of L-Amb may offer alternative regimens if renal toxicity becomes problematic (54). In addition, early consideration of PIIRS when CSF cultures have previously converted to negative may prevent unnecessary additional courses of amphotericin B.

Triazole antifungal drugs such as voriconazole, Posaconazole, and isavuconazole are potent inhibitors of cytochrome P450 3A4, playing important roles in metabolizing immunosuppressant drugs including cyclosporine, tacrolimus and sirolimus. Co-administration of such drugs with these immunosuppressants are likely to increase plasma levels of the respective immunosuppressants; conversely, discontinuation could lead to increased risks of rejection as immunosuppressant levels fall. For example elevated levels of cyclsporine and tacrolimus are potentially nephrotoxic and neurotoxic (99) and sirolimus, evorolimus and cyclosporine are associated with pulmonary toxicity (100). Thus, it is important to consider such interactions with appropriate drug level monitoring as indicated (101).

Other patient populations with pre-existing immune suppression also require attention to their immunosuppressive regimen to both facilitate fungal clearance and ameliorate the propensity to inflammatory sequelae. For example, almost 50% of patients in a recent prospective cohort of non-HIV patients with CM were found to be on various doses of corticosteroid therapy at the initial diagnosis (2). Patients on corticosteroid therapy for active inflammatory conditions are at risk for acquisition of CM and may require a modest reduction during the antifungal induction (102), but re-institution after CSF sterilization may be necessary both to manage the underlying condition and to minimize cryptococcal-related inflammation. Again, monitoring indicators of CSF inflammation as well as fungal cultures may add additional precision to the treatment algorithm. Another example of an immunosuppressant predisposing to cryptococcosis is the agent for multiple sclerosis, fingolimod, which predisposes to the fungus through the S1P receptor 3 of macrophages (103, 104). Fingolimod therapy is typically discontinued after cryptococcal infection, but in our experience, at least two of these MS patients developed PIIRS after discontinuation which was managed with antifungal and PCT corticosteroid therapy with good results. MS patients previously on fingolimod may need to reevaluate starting a different disease modulating therapy (DMT); however, this can likely be delayed until after the corticosteroid treatment for PIIRS has been tapered off or to a low dose.

## Summary

Cryptococcal disease, particularly that involving the CNS, presents an especially challenging condition to treat, complicated by the necessity to manage microbiological control and the all-too-frequent pathological inflammatory sequelae. In addition, co-morbid conditions both predisposing and a result of such infections must also be managed. Key to successful management is an understanding of the pathophysiology of the various syndromes, and the use of specific biomarkers and imaging to balance the two host-destructive features of the pathogen-inflammation parabola.

## Author contributions

KS, SA, JH, PC, SSh, SSe, KM, DH, BB, and PW all contributed substantially in the writing of this review. All authors contributed to the article and approved the submitted version.

## Funding

This work was supported in part by the intramural research program of NIAID.

## Conflict of interest

The authors declare that the research was conducted in the absence of any commercial or financial relationships that could be construed as a potential conflict of interest.

## Publisher's note

All claims expressed in this article are solely those of the authors and do not necessarily represent those of their affiliated organizations, or those of the publisher, the editors and the reviewers. Any product that may be evaluated in this article, or claim that may be made by its manufacturer, is not guaranteed or endorsed by the publisher.
